# Virtual Screening, Biological Evaluation, and 3D-QSAR Studies of New HIV-1 Entry Inhibitors That Function via the CD4 Primary Receptor

**DOI:** 10.3390/molecules23113036

**Published:** 2018-11-20

**Authors:** Chaozai Zhang, Huijun Zhang, Lina S. Huang, Siyu Zhu, Yan Xu, Xing-Quan Zhang, Robert T. Schooley, Xiaohong Yang, Ziwei Huang, Jing An

**Affiliations:** 1 Department of Medicine, Division of Infectious Diseases, School of Medicine, University of California San Diego, La Jolla, CA 92037, USA; chaozai2010@hotmail.com (C.Z.); zhanghj14@mails.tsinghua.edu.cn (H.Z.); lsh83@cornell.edu (L.S.H.); zhusy14@mails.tsinghua.edu.cn (S.Z.); xiz002@ucsd.edu (X.-Q.Z.); rschooley@ucsd.edu (R.T.S.); 2 School of Pharmaceutical Sciences, Jilin University, Changchun 130021, China; 3 School of Life Sciences, Tsinghua University, Beijing 100084, China; xyupstate@gmail.com; 4 College of Arts and Sciences, Cornell University, Ithaca, NY 14853, USA; 5 Nobel Institute of Biomedicine, Zhuhai 519000, Guangdong, China

**Keywords:** HIV-1, entry inhibitor, CD4, virtual screening, 3D-QSAR

## Abstract

Human immunodeficiency virus type 1 (HIV-1) is responsible for the majority of HIV infections worldwide, and we still lack a cure for this infection. Blocking the interaction of HIV-1 and its primary receptor CD4 is one strategy for identifying new anti-HIV-1 entry inhibitors. Here we report the discovery of a novel ligand that can inhibit HIV-1 entry and infection via CD4. Biological and computational analyses of this inhibitor and its analogs, using bioactivity evaluation, Rule of Five (RO5), comparative molecular field analysis (CoMFA)/comparative molecular similarity index analysis (CoMSIA) models, and three-dimensional quantitative structure-activity relationship (3D-QSAR), singled out compound **3** as a promising lead molecule for the further development of therapeutics targeting HIV-1 entry. Our study demonstrates an effective approach for employing structure-based, rational drug design techniques to identify novel antiviral compounds with interesting biological activities.

## 1. Introduction

Acquired immune deficiency syndrome (AIDS) is one of the major diseases of large public health impact. It is caused by HIV, and it represents the final stage of HIV infection [1]. One of the hallmarks of HIV infection is the selective destruction of CD4 T-cells [2]. Treatment for patients infected with HIV is usually antiretroviral therapy (ART), with double or triple drug combinations chosen from approved drugs [3,4]. The viral entry process is one of the most promising targets for the development of new anti-HIV drugs effective in the virus replication cycle for the long-term treatment of patients with AIDS [5,6]. Two types of HIV are recognized, HIV-1, which is responsible for the majority of HIV infections worldwide, and HIV type 2 (HIV-2), which causes the infection endemic in western Africa [7,8]. Here, our work mainly focuses on the worldwide infection caused by HIV-1.

CD4, as the primary receptor of the HIV-1 envelope glycol protein 120 (gp120), is critical for HIV-1 entry into host cells [9,10]. HIV-1 infection is initiated by the binding and attachment of gp120 to CD4 [11], which induces a series of conformational changes in gp120 that consequently expose its third variable (V3) loop for specific recognition of a coreceptor, either CCR5 or CXCR4 [12,13,14]. Coreceptor binding is the second obligatory event in HIV-1 entry, and it can specifically determine the tropism of HIV-1 infection [15,16]. Following the interaction of viral gp120 with its coreceptor, the gp120-gp41 complex undergoes a dramatic conformational change that leads to the formation of a trimeric hairpin structure of gp41, enabling the viral envelope to fuse with the host cell membrane. As a result, the viral capsid is released into the cytoplasm of the target host cell [17,18]. HIV-1 infection induces a progressive and quantitative decline in CD4^+^ T cell responses, and this finally leads to AIDS [19].

The interaction between CD4 and gp120 has been studied by mutagenesis and crystal structure determination to identify the CD4 residues that are implicated in gp120 binding [10,20,21]. The published data indicate that residues 29, 35, and 40–48 of CD4 are critical for the recognition of HIV-1 gp120 [22]. These residues form a region that is homologous to the second complementarity-determining region (CDR2) of immunoglobulin light chains [23]. In this interaction between CD4 and gp120, 63% of all interatomic contacts come from residues 40–48 of CD4, and Phe43 alone accounts for 23% of the total contacts [21]. 

These known interactions suggest that blocking the interaction of CD4 and gp120 could be a promising way to inhibit HIV-1 entry into its host cell. Three strategies are possible for the discovery of new anti-HIV drugs that work by blocking the interaction of CD4 and gp120 [24]. One is discovering new agents that can bind to gp120 and prevent the gp120/CD4 interaction. BMS (Bristol-Myers Squibb)-378806 (BMS-806) and NBD-556 are two representative small molecules [25]. BMS-378806 inhibits the interaction of gp120/CD4 and HIV infection at a nanomolar level [26]. Among the BMS family, BMS-663068, the prodrug of BMS-626529, was a more promising inhibitor that showed improved in vitro and pharmacokinetic properties and it is being assessed in a Phase III clinical trial [27,28]. Conversely, NBD-556 and its analogs, as CD4-mimics, were designed to target the Phe43 cavity of gp120 [29,30], thereby, inhibiting the gp120/CD4 interaction and HIV-1 infection. The initial lead compounds of this series, NBD-556 and NBD-557, were screened from a small molecule library and identified using a syncytium formation assay [31]. A further development of the NBD series, NBD11021, showed a more promising level of anti-HIV activity [32] when compared with other compounds presenting anti-HIV-1 activity, like the DMJ and JRC series compounds [33,34]. 

Because of the high rate of mutagenesis of gp120, drug resistance becomes one of the key problems to consider during the development of anti-HIV-1 therapeutic medicines [35], and it becomes unavoidable when searching for new potential leads targeting gp120. When drug resistance occurs, and especially cross-resistance, the ART drugs that share the same mechanism of action will be rendered ineffective. Thus, another strategy for the discovery of HIV-1 entry inhibitors, is to target CD4, the primary receptor of gp120. PIC 024-4 and PRO 2000 are naphthalene sulfonate polymers that bind to CD4, thereby blocking gp120/CD4 interaction with nanomolar affinities, and they showed anti-HIV-1 activity [36]; NSC13778 was another gp120/CD4 inhibitor that competed with gp120 for binding to the D1/D2 domain of the CD4 protein with a low micromolar anti-HIV-1 activity; NSC13778 was screened out using a cell-based anti-HIV-1 screen [37]. Another representative drug is ibalizumab, an FDA-approved ART drug for multi-drug resistant HIV. Ibalizumab is a humanized monoclonal antibody that binds to the D2 domain of CD4 with significant anti-HIV-1 activity and minor adverse effects [38,39]. Ibalizumab cannot directly block the gp120/CD4 interaction, but it induces conformational changes in the CD4-gp120 complex and prevents HIV entry [39]. 

The third strategy is downmodulation of CD4 and prevention of HIV entry. The representative compound is cyclotriazadisulfonamide (CADA), a small molecule that can decrease the amount of cell surface and intracellular CD4, thereby resulting in anti-HIV-1 activity [40]. 

In our manuscript, our strategy for discovery of new HIV-1 entry inhibitors was focused on a relatively conserved target, CD4, the primary receptor of gp120, which avoids much of any future concern about drug resistance [41]. A structure-based and computer-aided virtual screening (VS) approach, which searches chemical libraries to discover compounds that can interact with a protein structure with predicted binding affinities and modes, is one of the most powerful methods for identifying new inhibitors [42,43]. This approach is becoming increasingly important and effective for new drug discovery [44]. 

In this paper, we aimed to design novel non-peptide CD4 ligands based on the 3D structure of the CD4 protein. We conducted a VS study by targeting the critical amino acid residues on CD4 using Flex-Dock function of SYBYL-X program to identify compounds from the National Cancer Institute (NCI) structure diversity data set, a library consisting of 1974 compounds with diverse chemical structures that were predicted to bind the CD4 target. We collected 40 top-binding compounds predicted by VS and determined their actual CD4 antibody competitive binding affinities. Among the 40 compounds, compound **3** (NSC119915, 3-(4,5,6-trihydroxy-3-oxo-3H-xanthen-9-yl) propanoic acid) had the best CD4 competitive binding with an IC_50_ value of 45 μM. As CD4 is essential in the process of HIV-1 entry into host cells, HIV-1 entry inhibition assays were also performed for this most potent compound. HIV-1-infected groups treated with **3** showed inhibition of viral replication, indicated by a decrease in both green fluorescent protein (GFP) fluorescence and intracellular p24 level as compared with those of untreated groups. The IC_50_ values for these two measurements were 10 and 9 μM, respectively, indicating that **3** was able to suppress HIV-1 infection by inhibiting the viral entry process. 

Based on the discovery of **3**, we further examined 40 additional compounds of chemical structures that were similar or related to **3**, which were selected by the NCI database browser. This identified another new anti-HIV-1 compound **29**. A previously published paper by Robert J. Fisher et al. presented the result that **29**, generated from biological high-throughput screening using a robotic system, could bind to HIV-1 nucleocapsid-p7 protein (NC-p7), which is crucial in promoting reverse transcription [45,46] and could inhibit HIV-1 infection [45]; however, **3** was inactive or weakly active (0–49% protection) when evaluated in their anti-HIV-1 infection assays. Taken together, the results from our present study and from Fisher’s previous report indicate that **3** and **29** could act as potential dual-target HIV-1 inhibitors. CD4 competitive binding assays, in conjunction with 3D-QSAR studies, were performed on these compounds. The bioassay data were used to construct CoMFA and CoMSIA models with excellent parameters [47,48]. 

Taken together, the results from CD4 competitive binding and anti-HIV-1 infection assays showed that **3** is a promising new lead compound for the effective inhibition of HIV-1 entry via CD4. Our 3D-QSAR studies based on **3** also provide a deeper insight into the interaction of CD4 and its newly discovered ligands, and reliable 3D-QSAR models that can guide the optimization of the chemical structure of the lead compound. 

## 2. Results

### 2.1. Virtual Screening

**Figure 1:**
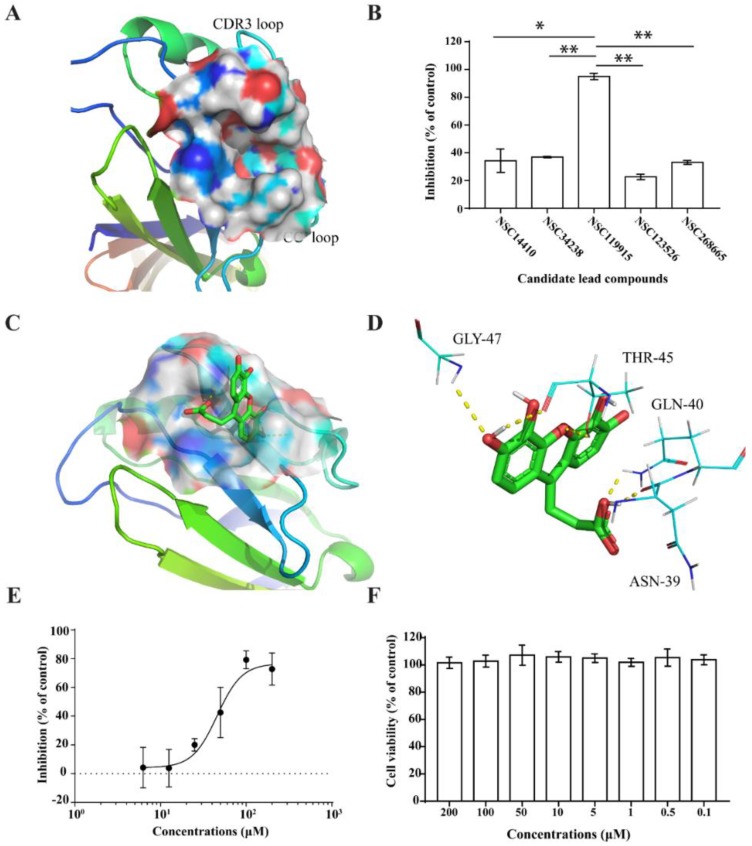
Binding pocket on CD4 surface (**A**) and compounds exhibited varying extents of inhibition of anti-human CD4 antibody binding to CD4 (**B**). The docking models of NSC119915 and CD4 receptor (**C**) and interaction of NSC119915 and CD4 (**D**). NSC119915 displayed dose-dependent inhibition (**E**) while not showing any cytotoxicity (**F**). * *p* value < 0.05, ** *p* value < 0.005.

The binding site of CD4, which is a hydrophobic cavity (Figure 1A) located near the critical amino acid sequences (40–48) for CD4 and gp120 interaction, was used as the target site to perform VS on the NCI diversity data set. Of the 1974 compounds docked, 40 top-score compounds were selected and analyzed for the interaction between the receptor and ligands. Five compounds showed CD4 binding activities and could form three or four H-bonds, which play an important role in ligand binding. These compounds were predicted to recognize the reported critical amino acid residues such as Asn39, Gln40, Thr45, and Gly47, which contribute to the interaction between CD4 and gp120.

### 2.2. Competitive CD4-Binding Compounds

We chose CD4-expressing SUP-T1 cells to perform the competitive CD4 binding assay. Non-labeled anti-human CD4 antibody (RPA-T4 clone, BioLegend, San Diego, CA, USA) was used as a positive control. We found that five compounds collected from the NCI diversity dataset had CD4 competitive binding activities (Table 1, Figure 1B).

Among the five compounds, **3** showed the best inhibition in this assay (Figure 1B). The docking model demonstrated that **3** could bind to the CD4 D1 CDR3-CC′ pocket [49] (Figure 1C) and form H-bond with residues Asn39, Thr45, and Gly47, which are critical in the interaction of CD4 and gp120 (Figure 1D). In addition, this compound also has Van der Waals (vdW) contacts with the CD4 receptor. The CD4 competitive binding inhibition of **3** at 100 μM was 94.94%, and the IC_50_ value was 45 μM (Figure 1E). 

We also performed a cell viability assay to test the potential cytotoxicity of **3**. In parallel with the competitive CD4 binding assay described above, the same densities of SUP-T1 cells were seeded and treated with the test compounds for the same duration. Compound **3** showed no cytotoxicity in the cell viability assay, even when used at a much higher concentration (200 μM) than that used in the competitive CD4 binding assays (Figure 1F). These data demonstrated that compound **3** is a CD4-binding lead compound of no cytotoxicity.

### 2.3. NSC119915 Displayed Potent Anti-HIV-1 Activity

**Figure 2:**
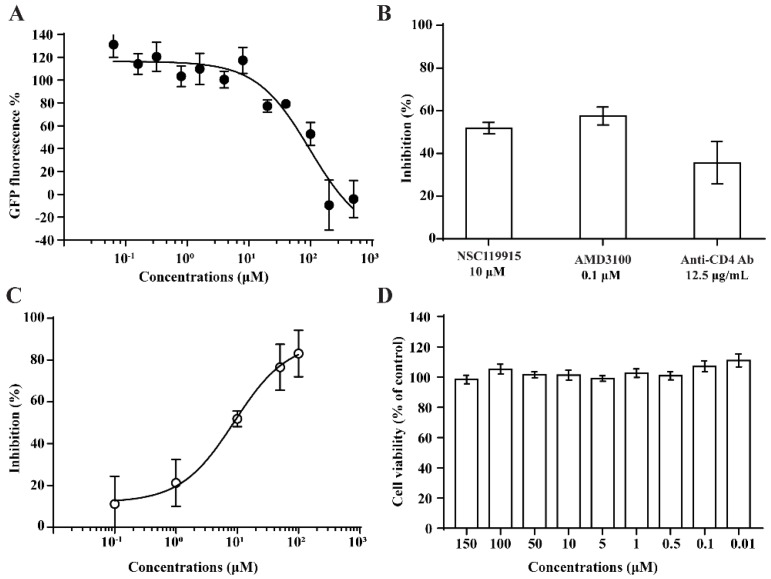
The decreased green fluorescent protein (GFP) fluorescence values indicate that the infection of HIV-1 was inhibited by **3** (**A**). **3** could significantly block HIV-1 entrance into PBMCs as the intercellular p24 level was decreased, AMD3100 and anti-human CD4 antibody used as positive controls (**B**). The HIV-1 entry inhibition assay showed dose-dependent results with an IC_50_ value of 9 μM (**C**) without a cytotoxicity effect (**D**).

We performed anti-HIV-1 infection and anti-HIV-1 entry assays by measuring GFP expression and intracellular p24 antigen level. For the anti-HIV-1 infection assays, the CEM-GFP reporter cell line was used, and HIV-1 infection induced a 10- to 1000-fold increase in the relative fluorescence of the cells [50]. Cells treated with **3** for 4–5 days showed decreased GFP fluorescence values in the HIV-1 infected group, with an IC_50_ value of 10 µM (Figure 2A). This indicated that **3** could inhibit HIV-1 infection. 

We also performed anti-HIV-1 entry assays to investigate the potential suppression by **3** of HIV-1 infection via its entry mechanism. Peripheral blood mononuclear cells (PBMCs) that were infected with HIV-1 (NL4-3 strain) for 24 h showed a decrease in their intracellular p24 antigen levels after treatment with **3**, as well as dose-dependent HIV-1 inhibition with an IC_50_ value of 9 μM (Figure 2C). The inhibition by a positive control compound AMD3100 at 0.1 μM was 57.6% (Figure 2B). These data further demonstrated that **3** acted as an HIV-1 entry inhibitor. 

### 2.4. NSC119915 Showed No Cytotoxicity in PBMCs

We employed a cell viability assay to test the potential cytotoxicity of **3**. In parallel with the anti-HIV-1 assay described above, the same densities of PBMCs were seeded and treated with the test compounds for the same duration. **3** showed no cytotoxicity in PBMCs, even when tested at a higher concentration (150 μM) than that used in the competitive CD4 binding assay (Figure 2D). 

### 2.5. Bioactivity Evaluations of Compounds Collected from Structural Similarity Screening

**Figure 3:**
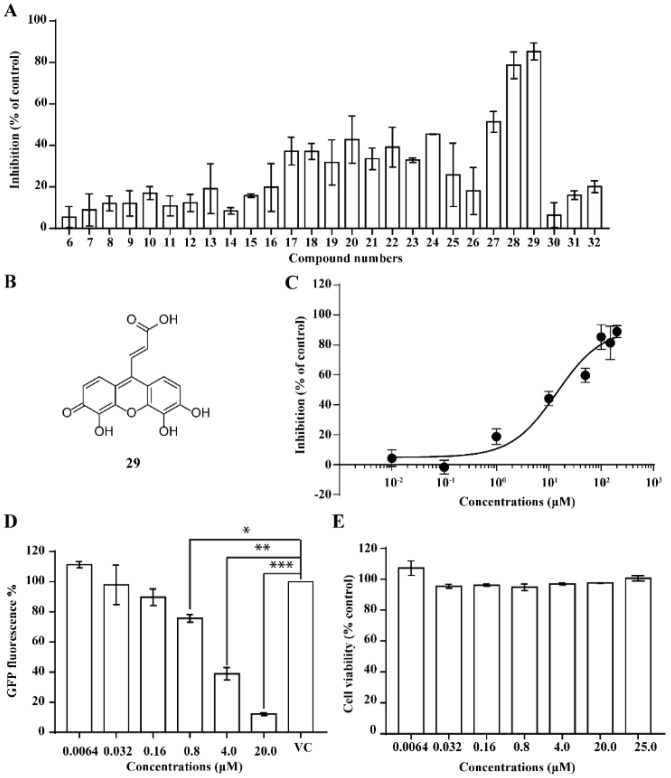
The competitive CD4 binding assay results of analogs of **3** (**A**). Among these analogs, **29** showed the best binding affinity with an IC_50_ value of 14 µM (**B**,**C**). **29** inhibited HIV-1 infection in a dose-dependent manner (**D**), and a CellTiter Blue assay of **29** showed a complete lack of cytotoxicity effect (**E**).

We performed a similarity search study using the Enhanced NCI Database Browser 2.2 to select 40 compounds from a total of 250,250 compounds with a Tanimoto index of above 90%, and obtained them from NCI. The Tanimoto index is a useful tool for 2D fragment-based similarity searching [51]. The competitive CD4 binding assay showed that compounds **17**, **18**, **19**, **20**, **21**, **22**, **23**, **24**, **25**, **27**, **28**, **29**, and **32** had better CD4 competitive binding affinities than other compounds, as their inhibitions at 100 µM were all above 20% (Figure 3A). The binding inhibition and compound structures are showed in Table 2 and Figure 3A. Among these compounds, **29** (Figure 3B) showed the best binding affinity when compared to the other compounds, and its chemical structure was quite similar to that of **3**. The binding inhibition of **29** in the anti-human CD4 antibody competitive binding assays was 85.29% at 100 μM, and the IC_50_ value was 14 μM (Figure 3C).

We also evaluated the anti-HIV-1 infection activity of **29** using the CEM-GFP reporter cell line. The GFP values were significantly lower for HIV-1 infected groups treated with **29** than the group without drug treatment, and the decrease of the GFP values were dose-dependent, indicating that **29** was an effective HIV-1 inhibitor identified by similarity search using **3** as a lead template. The IC_50_ value of **29** was 2 µM (Figure 3D), and **29** showed no cytotoxicity, as determined by the cell viability in the CEM-GFP cell line, when tested at the highest concentration of 25 µM (Figure 3E). These results confirmed that **29** is a potent HIV-1 inhibitor, and that its anti-HIV-1 activity was not due to cytotoxic effects on the host cells. These bioactivity data also provide evidence that **3** is a viable lead compound for developing new ligands to inhibit HIV-1 infection via CD4. 

Most of the compounds collected from NCI database using the similarity search approach share certain common substructures with **3**. These compounds were classified into four classes for further studies. The common structures of each classification and CD4 binding inhibition at 100 µM are shown in Table 2.

### 2.6. 3D Quantitative Structure-Activity Relationship (3D-QSAR) Analyses

**Figure 4:**
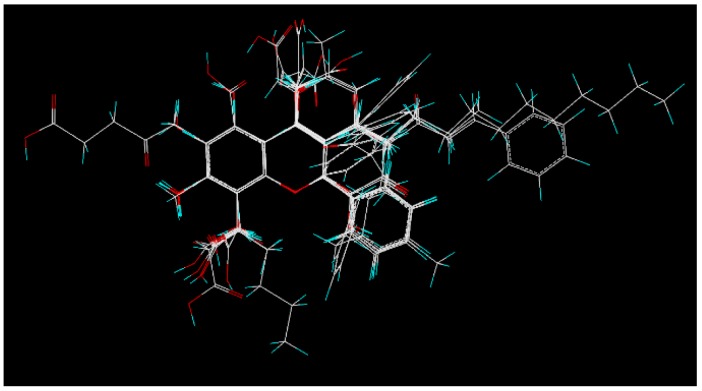
Alignment of all compounds in the training and test sets. Compound **3** was selected as the template for the alignment study, and the atoms of this compound were shown by different colors (C in white, H in cyan, and O in red).

The mechanism of the ligand-receptor interaction was investigated by performing a 3D-QSAR study, which is one of the most powerful approaches for guiding further lead optimization. A total of 27 compounds collected from similarity search based on **3** were divided into training and test sets, containing of **19** and **8** compounds, respectively, for 3D-QSAR studies. Compound **31** was discarded for its bad alignment with the same substructure of other compounds listed in Table 2. The training set was used to generate 3D-QSAR models, while the test set was employed to validate the quality of the models. In the alignment step, all compounds of the training and test sets were well aligned with the same substructure. The alignment result is shown in Figure 4.

CoMFA and CoMSIA studies were performed based on the molecular alignment, as described in the methods. CoMFA calculates the steric and electrostatic properties, whereas CoMSIA calculates similarity indices in the space surrounding each of the molecules in the dataset [47,48]. The statistical results generated from the leave-one-out (LOO) cross validation of the CoMFA/CoMSIA models for CD4 binding activity are shown in Table 3, which lists the partial least squares (PLS) statistical results of the CoMFA/CoMSIA with a determined optimum number of components (ONC), cross-validated correlation coefficient (q^2^), non-cross-validation correlation coefficient (r^2^), standard error of the estimate (SEE) and the F value. The CoMFA model is derived from three components, showed a q^2^ of 0.624, a r^2^ of 0.961, a SEE of 0.145 and an F value of 141.287, and the CoMSIA model derived from 4 components, showed a q^2^ of 0.732, a r^2^ of 0.973, a SEE of 0.124, and an F value of 127.654. In further cross-validation of the CoMFA/CoMSIA models, the CoMFA mean q^2^ values of the leave-many-out (LMO_10_ and LMO_5_) cross-validation were 0.630 and 0.561, respectively. The average r^2^ and SEE of 100 runs of bootstrapping analysis were 0.969 and 0.122. The CoMSIA mean q^2^ values of LMO_10_ and LMO_5_ cross-validation were 0.736 and 0.685, respectively. The average r^2^ and SEE of the 100 runs of bootstrapping analysis were 0.985 and 0.085. The values of the bootstrap r^2^ confirmed further that the CoMFA/CoMSIA models in our studies were both good models for 3D-QSAR studies. All these parameters indicated a satisfactory internal predictive ability of the model.

**Figure 5:**
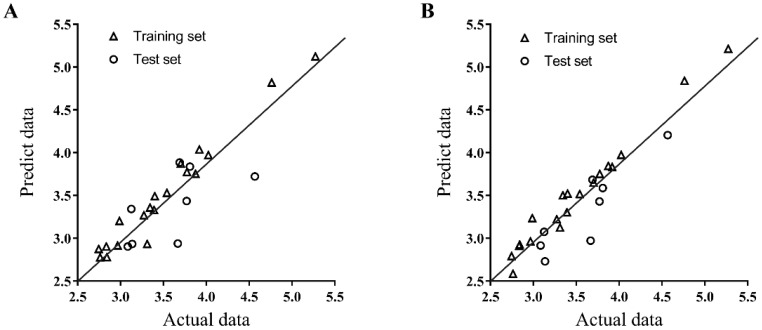
Plots of predicted inhibition values versus the actual values for the training set and the test set compounds from the CoMFA (**A**) and CoMSIA (**B**) models. The experimental and predicted data of CD4 competitive binding inhibition was presented as -ln.

For a further evaluation study, the CoMFA/CoMSIA models were employed to predict the activity of the test set, and the result was compared with the experiment data. The experimental and predicted data with the residuals are listed in Table 4, and the relationship between the experimental and predicted activity values of the training set and the test set are depicted in Figure 5. The predicted activity values were in good agreement with the experimental data. The CoMSIA model was better than CoMFA model when compared with the PLS parameters and residuals listed in Table 3 and Table 4. Therefore, we chose the CoMSIA model for further discussion of the 3D-QSAR results. 

**Figure 6:**
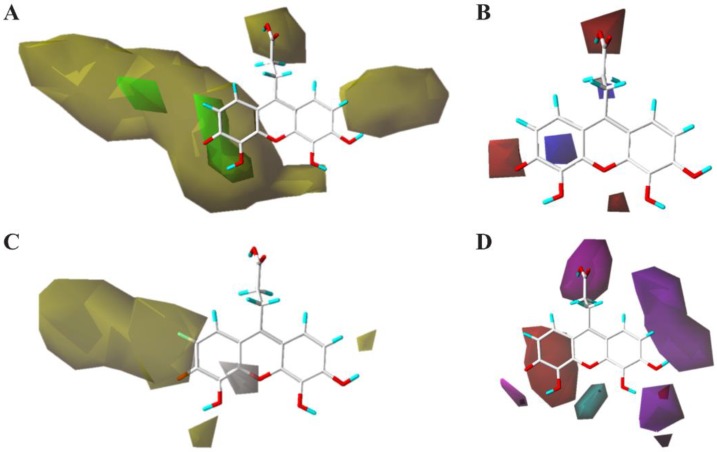
The contour maps of 3D-QSAR model show: (**A**) the steric bulk favored (green) and steric bulk disfavored (yellow) regions; (**B**) positive charge desirable (blue) and negative charge desirable (red) regions; (**C**) hydrophobicity desirable (yellow) and hydrophobicity undesirable (white) regions; (**D**) hydrogen bond donor desirable (cyan), hydrogen bond donor undesirable (purple), hydrogen bond acceptor desirable (magenta), and hydrogen bond acceptor undesirable (red) regions.

To visualize the fields effects of the CoMSIA model, 3D contour maps in Figure 6 were generated from the SYBYL-X program, the data was transformed using from % inhibition at 100 micromolar concentration, and the transformed data was named as pIn. **3** was selected as a reference and overlaid in the maps, as it is the most active compound in the training and test sets. In the CoMSIA contour maps, various (steric, electrostatic, hydrophobic, hydrogen bond donor and hydrogen bond acceptor) characters of the compounds were collected, and they contributed 22.3%, 10.2%, 23.0%, 22.7%, and 21.8%, respectively, to the interaction of compounds listed in Table 4. 

The yellow contour of the CoMSIA steric contour map (Figure 6A) near positions 6 and 7 of the 3-oxo-3H-xanthen ring shows that these positions are sterically unfavorable regions (i.e., compound **32**), and propanoic acid at 3-position of 3-oxo-3H-xanthene ring is also an unfavorable substituent group. This steric contour map agreed with the docking model of **3** shown in Figure 1A, which demonstrated that these positions can interact with residues in the cavity of CD4. These observations indicated that modifications of these compounds with lager substituent groups should be avoided at these positions. The electrostatic contour map of the CoMSIA model (Figure 6B) showed that the electronegative groups were favored near the 2, 5 positions of the 3-oxo-3H-xanthene ring, and another electronegative group was favored near the carboxyl of the propanoic acid substituent. This was consistent with the increased activity of compounds **24**–**27**, compared with compounds **14**–**16**. The electropositive region was found in the cyclohexa-2,5-dien-1-one region. In Figure 6C, the yellow contour indicates a favorable hydrophobic interaction region near the 3, 4, 6, 7 positions of the 3-oxo-3H-xanthene ring. These contours can explain the decreases in the activity of compounds **32** and **24**–**27** when compared with compound **3**. The white contour showed an unfavorable hydrophobic interaction region near the 4 position of the 3-oxo-3H-xanthene ring. Figure 6D shows the contour map of the hydrogen bond donor/acceptor. The magenta contour represents a desirable hydrogen bond acceptor and it agreed with the interaction model shown in Figure 1D. The propionyloxy group of **3** can form three hydrogen bonds with the Phe26, Asn39, and Gln40 residues of CD4, and Asn39 and Gln40 are both critical residues in the procedure of the interaction between CD4 and gp120. The magenta contour near the 1 and 2 position represents an undesirable hydrogen bond, which agreed with the interaction model for CD4 and gp120 where the H atoms at the 1 and 2 positions did not contribute to forming any hydrogen bond with the receptor. 

Based on the analysis of CoMSIA contour maps, our bioassay data agree well with the 3D-QSAR model, and provide useful information for the new activity of predictable compounds that are designed from the template **3**. Furthermore, based on the bioassay data of **3** and **29**, the contour maps can help design anti-HIV-1 entry inhibitors from the template **3**. 

## 3. Discussion

CD4 is critical in the process of HIV-1 invasion into its host cells, and blocking the interaction of CD4 and gp120 can significantly inhibit HIV-1 entry into host cells [52]. We investigated new HIV-1 entry inhibitors via CD4 by employing a structure-based virtual screening approach to dock compounds from the NCI diversity data set to some critical residues of the cavity of CD4. Among the docked top-score compounds generated from this virtual screening study, **3** showed strong competitive binding affinity with CD4, and good HIV-1 entry inhibition activity without showing cytotoxicity. The calculated properties of **3** showed that: (1) there are four hydrogen bond donors; (2) there are six hydrogen bond acceptors; (3) the molecular weight is 316.27 Daltons; (4) the logP is −1.335; and (5) the number of rotatable bonds is three. All five properties of **3** meet the Lipinski’s rule of five (i.e., RO5, specifically MWT < 500, logP < 5, H-bond donors < 5, H-bond acceptors < 10, rotatable bond < 10) [53]. Combined with the bioassay data, **3** was found to be a drug-like compound and a viable lead with good bioactivity and no cytotoxicity. Considering the characteristics of its molecular structure, some previous publications have indicated that **3** at a high concentration (100 μM) could inhibit the growth of various cancer cell lines by increasing the intracellular reactive oxygen species (ROS) level [54,55]. Based on our bioassay data, especially our PBMC cell viability assay results, **3** did not show an ability to affect cell growth at the concentrations (≤150 μM) used in our bioassays (Figure 2D). This finding demonstrated that the ROS mechanism may not be the reason for why **3** acts as an HIV-1 inhibitor. The chemical structure of **3** also suggests that additional structurally similar compounds can be discovered that can act as potent CD4-binding ligands. Our data indicated that **3** could be a promising compound for the development of new HIV-1 entry inhibitors that act via CD4, and that other structurally similar compounds can be discovered based on the chemical structure of **3**. According to the data published by Robert J. Fisher et al. [45], **29** could also bind to NC-p7, and contribute to inhibition of HIV-1 infection. Although **3** was regarded as an inactive or less effective (0–49% protection) compound when evaluated in their anti-HIV-1 assay, our data revealed another function of **3** and **29**, as these two compounds could also bind to CD4 (the primary receptor of gp120), and may therefore act as potential dual-targeting agents to inhibit HIV-1 infection. Support for this idea was provided by two advanced anti-HIV-1 infection assays: recording the fluorescence value change of infected CEM-GFP cells, and determining the intracellular p24 levels in the PBMCs. The relationship of ligand structure and CD4 binding activity was investigated in more detail with the 3D-QSAR method. Twenty-seven compounds generated by a similarity search with the lead compound **3** were used to build 3D-QSAR models. CoMFA and CoMSIA models obtained based on ligand alignment both had high q^2^ and r^2^ values (both greater than 0.5) and small standard errors of estimates. The models were also validated by LMO cross-validation, bootstrapping, and activity prediction of the test set. All the validations confirmed the models as being reliable and with a high predictive ability. The chemical interpretation of the contour maps generated by CoMSIA agreed well with the changes in the chemical structures and the bioactivity values of the compounds. We also investigated that all our compounds that showed a greater than 30% level of inhibition, using the PubChem database and online literature searches for any reported bioactivity and targets. The results were complicated, as compounds **3** and **29** had been reported as being active in several bioassays and multitargets; the active results and targets (Hsp70; RAPGEF4; GAPDH; RNASEH1) were fewer for **29** than for **3**. However, other compounds had even fewer or no reported bioactivities. Some of these reported compounds were focused on anticancer screening or antimicrobial assays without targeting a specific receptor. Since the pan-assay interference compounds (PAINS) filter was first reported, it has become a common component of the triage process in biological screening [56]. Based on the PAINS filter results, some of our compounds (**3**, **28** and **29**) failed to pass, and other compounds that came from similarity searches and that were used in establishing 3D-QSAR models all passed the filter (Table 5). Recently, a large-scale analysis of PAINS alerts was reported, indicating that computational PAINS filters may inappropriately flag some compound classes, creating the potential for incorrect prediction. In fact, 87 Food and Drug Administration (FDA) approved drugs contained PAINS alerts. The current recommendation is that conclusions should be drawn only after conducting orthogonal experiments. [57]. Based on theory, published large-scale analysis results, and our results reported here, some of our compounds could pass a PAINS filter and show bioactivity based on a compound that failed to pass the filter. This leads us to consider the advantage of our 3D-QSAR models, which could help future studies to avoid failing the PAINS filter, and that could provide higher specificity and lower risk based mainly on class I and class II compounds (Table 2).

In conclusion, the results from virtual screening, CD4 competitive binding assays, and HIV-1 entry inhibition assays, together with the 3D-QSAR models, can guide the use of molecular databases for the discovery of new CD4 ligands that inhibit HIV-1 entry. The lead compound **3** is an effective template for the identification of additional HIV-1 entry inhibitors. The mechanistic study using the 3D-QSAR method provided valuable insights into the structure-activity relationship of **3** and its analogs. Our 3D-QSAR models can also be used to predict the activity of new small molecules designed based on **3** prior to biological testing, making these models be a valuable tool for optimizing lead compounds with higher activities.

## 4. Materials and Methods

### 4.1. Molecular Modeling and Virtual Screening

The molecular modeling and virtual screening were performed using the Surflex-Dock module implemented in the SYBYL-X 2.1 program (Tripos Inc., St. Louis, MO, USA). Before the docking procedures, water molecules and other ligands were removed from the crystal structure (PDB: 1GC1 [21]), and energy minimization was performed. The NCI diversity dataset, which contains 1,974 diversity compound structures, was selected for the virtual screening study and prepared using the preparation protocol of Surflex-for-searching in the ligand structure preparation tool. The protomol (an idealized active site ligand to generate putative poses of molecules [58]) was generated, selecting the critical amino acids (residues 29, 35, 40–49) in the interaction of CD4 and gp120 [21,59]. In the docking procedure, the minimization was performed pre-dock and post-dock. A total of 20 poses were generated from each docked ligand. Forty compounds of top-ranking total scores were selected for further bioassays. All compounds were ordered from the Division of Cancer Treatment & Diagnosis (DCTD) of the National Cancer Institute (NCI) (website: https://dtp.cancer.gov/organization/dscb/obtaining/vialed.htm) [60].

### 4.2. CD4 Competitive Binding Assay

The SUP-T1 cells were cultured in RPMI1640 medium supplemented with 10% (*v/v*) fetal bovine serum (FBS), 100 IU penicillin, 0.1 mg/mL streptomycin, and 2 mM L-glutamine. After collecting and washing twice with fluorescence activated cell sorting (FACS) buffer (0.5% BSA, 0.05% sodium azide in PBS) by centrifugation, SUP-T1 cells were seeded in a 96-well v-bottom plate at 5 × 10^5^ cells/well. The supernatant was discarded by centrifugation and the cell pellet was resuspended with 50 μL various 2× concentrations of test compounds and 50 μL fluorescein isothiocyanate (FITC) labeled anti-human CD4 antibody (RPA-T4 clone, BioLegend, San Diego, CA, USA). The plate was incubated for 40 min on ice. After incubation, the cells were washed twice with FACS buffer by centrifugation, and then resuspended with the assay buffer. The FACS buffer was added at a volume of 50 μL per well. The fluorescence (485_EX_/528_EM_) was recorded using a Synergy II plate reader (BioTek Instruments, Inc., Winooski, VT, USA). Experimental data were generated in duplicate each time, and from at least three independent experiments. The mean values of fluorescence were normalized and expressed as a percentage of the control group values. Binding curves and IC_50_ values were calculated by GraphPad Prism 7 (GraphPad Software Inc., La Jolla, CA, USA) and presented as the mean ± SEM.

### 4.3. Peripheral Blood Mononuclear Cell (PBMC) Isolation

Blood samples were collected from healthy donors and diluted with an equal amount of 1× PBS. Approximately 15 mL Ficoll was transferred to a sterile 50 mL tube, and then overlaid with 15 mL diluted blood. After centrifugation (Centrifuge 5810R, Eppendorf, Hamburg, Germany) at 1800 rpm for 30 min at room temperature, the PBMCs were obtained and washed twice with 40 mL 1× PBS by centrifugation (1100 rpm, 10 min for each time) at room temperature. Cell pellets were resuspended in 50 mL RPMI 1640 supplemented with 10% FBS and 100 μg/mL Phytohemagglutinin (PHA, Sigma-Aldrich, St. Louis, MO, USA) and cultured for 24 h at 37 °C with 5% CO_2_. Thereafter, PBMCs were continuously cultured in RPMI 1640 medium containing 10% FBS and 5 IU/mL human Interleukin-2 (hIL-2, Roche Diagnostics, Basel, Switzerland).

### 4.4. Anti-HIV-1 Infection Assay

The CEM-GFP cells were obtained from the NIH AIDS program (NIH AIDS Reagent Program, Division of AIDS, NIAID, NIH: CEM-GFP Cells from Dr. Jacques Corbeil [50]) and cultured in RPMI 1640 medium containing 10% FBS, 1% penicillin-streptomycin (Life Technologies, Carlsbad, CA, USA), 1% 200 mM L-glutamine (Mediatech-VWR, Aurora, CO, USA), and 500 µg/mL G418 (BioPioneer Inc, San Diego, CA, USA). After collecting and counting, the CEM-GFP cells were infected with HIV-1 NL4-3 strain (MOI 0.01) at 37 °C for 2–3 h, cells were then washed twice with PBS, and 10^5^ cells/well were seeded into a 96-well plate in the presence of different concentrations of test compounds. After culturing at 37 °C with 5% CO_2_ for 4 or 5 days, the GFP fluorescence (485_EX_/528_EM_) was measured using a Synergy II microplate reader (BioTek Instruments, Inc., Winooski, VT, USA).

### 4.5. Anti-HIV-1 Entry Assay

The Anti-HIV-1 entry assay was performed by measuring intercellular p24 antigen. Before the infection, PBMCs (2.5 × 10^6^ cells/mL) were added into 96-well plates at 100 μL/well, then co-incubated with compounds of various concentrations for 2 h. PBMCs were then washed twice with PBS and infected with the NL4-3 strain of HIV-1 at an MOI of 0.02 for 2 h at 37 °C with 5% CO_2_ (CO_2_ incubator, Eppendorf, Hamburg, Germany). After two washes with PBS (1200 rpm, 20 °C, 5 min), the cells were re-suspended in 100 µL complete medium and incubated for 24 h. Subsequently, the PBMCs were washed and resuspended in 100 µL/well lysis buffer containing 1% protease inhibitor cocktail (Sigma-Aldrich, St. Louis, MO, USA). The intercellular p24 antigen was measured according to the protocol provided with the HIV-1 ELISA kit (PerkinElmer Inc. Waltham, MA, USA).

### 4.6. PBMC Viability Assay

In addition to the anti-HIV assays described above, the same numbers of PBMCs were seeded and treated with the test compounds for the same periods. After a 24 h incubation at 37 °C, the cell viability was determined using the CellTiter-Blue (Promega Co., Madison, WI, USA) cell viability assay. The fluorescence (560_EX_/590_EM_) was recorded using a Synergy II microplate reader.

### 4.7. Compound Structural Similarity Search

For more chemical structures similar to compounds that were identified from the NCI diversity database, we conducted a similarity search study using the enhanced NCI database browser 2.2 (https://cactus.nci.nih.gov/ncidb2.2/) [61]. We obtained 40 more compounds with >90% Tanimoto index (which is superior to the Euclidean distance in 2D-fragment based similarity searching) from the NCI. Subsequently, competitive CD4 binding assays were performed to identify compounds that can bind to CD4.

### 4.8. 3D-QSAR Study

The 27 compounds involved in 3D-QSAR study were generated from the structure similarity search listed in Table 2 and the lead compound **3** in Table 1. Molecules that had no bioactivity and did not share a common scaffold were discarded. All ligands’ 3D structures were constructed using the Sketch Molecule function in SYBYL-X software. Gasteiger-Hückel charges were employed to calculate the partial atomic charges, and a TRIPOS force field was used for energy minimization with a convergence criterion of 0.05 kcal/mol·Å. The total compounds were divided into a training set and a test set.

Subsequently, molecular alignment was performed to obtain valid and reliable 3D-QSAR models [62]. In this study, **3**, which had the most potent inhibitory activity was chosen as the template molecule to perform the molecular alignment by using the database align function in the SYBYL-X program. Based on the molecular alignment, CoMFA/CoMSIA models were developed for the aligned molecular data set. In this study, five molecular fields were calculated: steric, electrostatic, hydrophobic, hydrogen bond donor, and hydrogen bond acceptor. CoMSIA molecular fields were calculated using a sp^3^ carbon probe atom carrying a charge of +1.0, and an attenuation factor α of 0.3 using default settings in SYBYL.

To generate statistically significant 3D-QSAR models, a PLS regression was carried out. The LOO cross-validation analysis was performed to determine the ONC and q^2^. After that, non-cross-validation analysis was performed with a threshold column filtering of 2.0 kcal/mol to generate the optimally PLS regression models for CoMFA/CoMSIA. The r^2^, SEE, and F ratio between the variances of experimental and predicted activity values were obtained.

To further evaluate the statistical significance of the derived models, additional cross-validation analyses were performed using groups in the training set. The CoMFA and CoMSIA models were further analyzed by additional rigorous statistical cross-validation, using 10 and five groups in the training set, and each cross-validation process was repeated 25 times. Subsequently, a bootstrapping analysis for 100 runs was performed to measure the bias of the original calculations. In addition, the external predictive ability of the CoMFA/CoMSIA model was assessed by prediction the activity of the test set and compared with the experiment data. After the evaluation of 3D-QSAR model, contour maps of all fields were constructed with default values of 80% favored and 20% disfavored contributions.

### 4.9. Data and Statistical Analysis

Statistical analysis was performed using a one-way ANOVA (GraphPad Software: GraphPad Prism 7 for Windows, GraphPad Software Inc., La Jolla, CA, USA). Average values were expressed as mean ± SD or SEM, n > 3. The results of calcium influxes/effluxes and Western blots were representatives of at least of three independent experiments. A P value less than 0.05 was considered statistically significant. * *p* value < 0.05, ** *p* value < 0.005, *** *p* value < 0.0005.

## Figures and Tables

**Table 1:** The structures and inhibition of five bioactive compounds from the National Cancer Institute (NCI) dataset.

No.	NSC No.	Structure	Inhibition at 100 μM (%)
**1**	14410	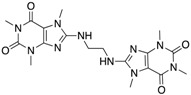	34.22
**2**	34238	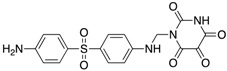	36.86
**3**	119915	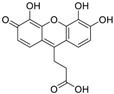	94.94
**4**	123526	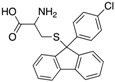	22.61
**5**	268665	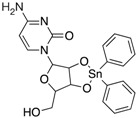	33.09

**Table 2:** The structures and inhibition data of compounds generated from the NCI similarity search.

Class I:								
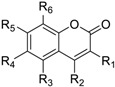								
**No.**	**NSC No.**	**R_1_**	**R_2_**	**R_3_**	**R_4_**	**R_5_**	**R_6_**	**Inhibition at 100 μM (%)**
**6**	5302	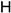	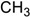	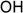	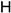	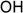	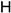	5.64
**7**	11840	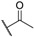	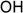	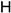	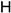	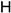	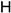	8.87
**8**	29117	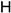	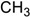	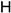	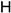	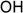	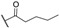	12.04
**9**	45749	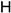	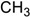	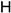	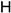	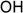	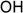	6.40
**10**	65625	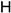	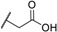	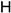	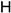	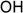	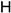	16.98
**11**	72276	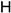	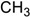	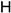	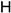	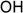	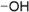	10.83
**12**	100977	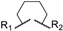	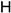		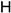	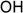	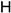	5.27
**13**	118660	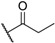	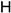	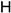	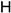	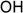	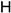	11.80
**14**	251156	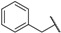	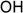	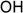	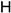	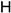	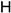	8.47
**15**	289346	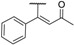	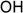	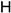	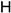	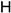	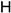	15.80
**16**	372920	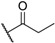	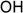	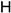	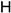	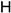	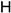	19.76
**17**	372922	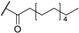	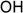	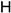	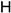	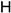	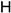	37.27
**18**	372923	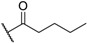	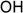	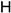	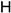	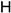	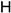	37.10
**19**	642907	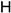	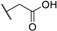	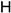	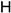	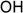	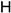	31.77
**20**	649799	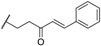	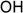	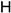	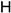	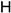	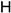	42.79
Class II:								
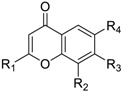								
**No.**	**NSC No.**	**R_1_**	**R_2_**	**R_3_**	**R_4_**	**Inhibition at 100 μM (%)**		
**21**	347512	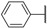	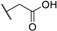	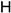	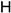	33.51		
**22**	354633	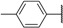	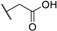	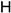	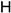	39.17		
**23**	358315	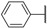	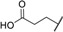	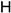	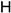	32.87		
**24**	361582	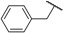	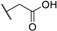	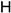	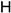	45.42		
**25**	361583	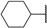	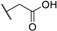	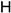	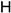	25.79		
**26**	362083	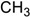	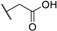	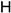	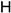	18.10		
**27**	383452	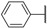	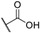	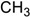	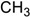	51.37		
Class III:								
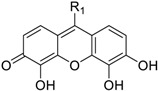								
**No.**	**NSC No.**	**R_1_**	**Inhibition at 100 μM (%)**					
**28**	119911	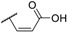	78.62					
**29**	158917	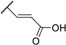	85.29					
Class IV:								
Other compounds								
**No.**	**NSC No.**	**Structure**	**Inhibition at 100 μM (%)**					
**30**	53584	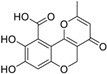	6.48					
**31**	107022	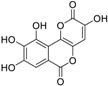	16.02					
**32**	61851	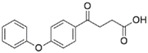	20.09					

**Table 3:** Statistical results of the CoMFA/CoMSIA models.

PLS Statistics	CoMFA	CoMSIA
Optimum number of components (ONC)	3	4
q^2^	0.624	0.732
r^2^	0.961	0.973
SEE	0.145	0.124
F	123.938	127.654
Field contribution%		
Steric	49.5	22.3
Electrostatic	50.5	10.2
Hydrophobic		23.0
Hydrogen bond donor		22.7
Hydrogen bond acceptor		21.8

**Table 4:** Experimental and predicted CD4 competitive binding inhibition of compounds in training and test sets by CoMFA/CoMSIA.

No	PInhibition ^a^ (Experimental Data)	CoMFA		CoMSIA	
		PInhibition ^a^ (Predicted Data)	Residual	PInhibition ^a^ (Predicted Data)	Residual
**3**	5.273	5.124	0.149	5.215	0.058
**6**	2.762	2.777	−0.015	2.586	0.176
**7**	2.988	3.203	−0.215	3.236	−0.248
**8** ^b^	3.136	2.934	0.202	2.731	0.405
**9**	2.835	2.905	−0.07	2.911	−0.076
**10**	3.311	2.934	0.377	3.125	0.186
**11** ^b^	3.084	2.905	0.179	2.915	0.169
**12**	2.745	2.876	−0.131	2.793	−0.048
**13** ^b^	3.126	3.342	−0.216	3.075	0.051
**14**	2.966	2.916	0.05	2.965	0.001
**15**	3.273	3.269	0.004	3.223	0.050
**16**	3.391	3.330	0.061	3.304	0.087
**17**	3.774	3.775	−0.001	3.752	0.022
**18** ^b^	3.771	3.436	0.335	3.430	0.341
**19** ^b^	3.668	2.940	0.728	2.972	0.696
**20**	3.874	3.755	0.119	3.846	0.028
**21**	3.702	3.872	−0.170	3.651	0.051
**22** ^b^	3.809	3.837	−0.028	3.587	0.222
**23** ^b^	3.690	3.886	−0.196	3.684	0.006
**24**	3.920	4.037	−0.117	3.834	0.086
**25**	3.541	3.531	0.010	3.517	0.024
**26**	3.344	3.360	−0.016	3.503	−0.159
**27**	4.024	3.972	0.052	3.974	0.050
**28** ^b^	4.566	3.722	0.844	4.206	0.360
**29**	4.763	4.819	−0.056	4.841	−0.078
**30**	2.841	2.780	0.061	2.928	−0.087
**32**	3.400	3.493	−0.093	3.523	−0.123

^a^ The experimental and predicted data of CD4 competitive binding inhibition was presented as -ln. ^b^ Test set.

**Table 5:** The investigation results based on PubChem database and the PAINS filter.

No.	NSC No.	Bioassay (Active) ^a^	PAINS Filter ^b^
**17**	372922	1 (anticancer screen)	PASS
**18**	372923	4 (antimicrobial assay; histone lysine methyltransferase G9a inhibitor; SWI/SNF chromatin remodeling complex inhibitor)	PASS
**19**	642907	7 (Grb2; HRAR1; p56 lck tyrosine kinase; Fyn protein kinase; phospholipase C gamma	PASS
**20**	649799	NONE	PASS
**21**	347512	5 (all for anti-cancer screens in mice using different models)	PASS
**22**	354633	1 (anticancer drug screen. Data for tumor model P388 Leukemia in mice)	PASS
**23**	358315	NONE	PASS
**24**	361582	NONE	PASS
**25**	361583	NONE	PASS
**26**	362083	NONE	PASS
**27**	383452	4 (anticancer drug screen in mice, fructose-bisphosphate aldolase inhibitor)	PASS

^a^ The contents in the bioassay column are presented as “reported active bioassay number (bioassays names or functions)”, if none was reported, a “NONE” presents. ^b^ PAINS was evaluated using the function of the PAINS filter in SYBYL-X. A “PASS” means a compound passes through the PAINS filter.
